# Toward Detection of FeH^+^ in the Interstellar Medium: Infrared Multiple Photon Dissociation Spectroscopy of Ar_2_FeH^+^

**DOI:** 10.1021/acs.jpclett.2c01511

**Published:** 2022-06-21

**Authors:** Shan Jin, Jakob Heller, Christian van der Linde, Milan Ončák, Martin K. Beyer

**Affiliations:** Institut für Ionenphysik und Angewandte Physik, Universität Innsbruck, Technikerstrasse 25, 6020 Innsbruck, Austria

## Abstract

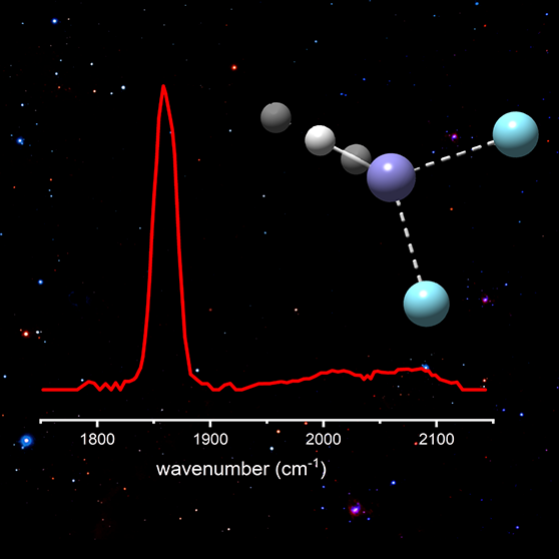

The iron hydride molecular cation FeH^+^ is expected to be present in the interstellar medium. Because of the lack of laboratory data, it is yet to be identified in spectrally resolved astronomic observations. As a benchmark for computational predictions and to guide an experimental search for the ro-vibrational lines of FeH^+^, we performed infrared multiple photon dissociation (IRMPD) spectroscopy of FeH^+^ tagged with two argon atoms. The Fe–H stretching mode in Ar_2_FeH^+^ is observed at 1860 cm^–1^. Combination bands of the Fe–H stretch with the two Fe–H bending and the asymmetric Fe–Ar stretching modes are observed at 2012 cm^–1^, 2054 cm^–1^, and 2078 cm^–1^. Quantum chemical calculations show that the molecule has C_2v_ symmetry. The Ar–Fe–Ar bending mode at 46 cm^–1^ is significantly populated at the temperature of the experiment, causing thermal broadening of the Fe–H stretch and its redshift with increasing internal energy.

Iron is among the most abundant elements in the universe, but little atomic iron is seen in the interstellar medium (ISM).^1−3^ Proposed sinks of iron include iron-containing nanoparticles^2^ and iron pseudocarbynes^1^ as well as molecular species like FeCN, which has recently been detected in the interstellar medium,^3^ and evidence has been found for FeO.^4^ Although hydrogen is by far the most abundant element, FeH or FeH^+^ have not been observed in the ISM.^5^ Absorption bands assigned to FeH have been identified in brown dwarfs^6^ and in the solar spectrum.^7,8^ A recent search for FeH in circumstellar envelopes, however, was not successful.^9^ The identification of FeH^+^ via observational data is so far not possible since no laboratory data are available on the spectroscopy of this diatomic molecular ion in any region of the electromagnetic spectrum despite the high priority assigned to laboratory spectra of FeH^+^ some 40 years ago.^10^

Experimental work has so far been limited to collisional activation and reactivity studies.^11−15^ Elkind and Armentrout obtained a 0 K bond dissociation energy of *D*_0_(Fe^+^–H) = 205 ± 6 kJ mol^–1^ with Fe^+^ in its ^6^D ground state.^13^ Thermochemical values on the neutral FeH molecule were obtained from hydride transfer reactions to Fe^+^.^14,15^ Irikura, Goddard, and Beauchamp calculated the rate of FeH^+^ formation via radiative association of H to Fe^+^ to be 5 × 10^–22^ cm^3^ s^–1^, concluding that this rate is too low for FeH^+^ to play a significant role in interstellar chemistry.^16^ However, other routes to its formation may be available such as the exchange of a weakly bound ligand X against the strongly bound and abundant H atom, reaction 1. Candidates for X are H_2_ and H_2_O, abundant molecules in interstellar space that should more efficiently attach via radiative association to Fe^+^, reaction 2:
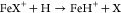
1
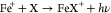
2

Among earlier theoretical works,^17−21^ the 1991 publication by Langhoff and Bauschlicher provides probably the most reliable 0 K bond dissociation energy, *D*_0_(Fe^+^–H) = 210 kJ mol^–1^,^20^ which agrees within error limits with the best experimental value by the Armentrout group.^13^ A recent, extensive study of Cheng and DeYonker^5^ largely confirmed the electronic structure found by Langhoff and Bauschlicher^20^ and provided spectroscopic constants and excitation energies of the electronic ground state and seven low-lying bound excited states of FeH^+^. The fundamental vibrational transition of FeH^+^ is predicted to lie at ν̃_0_ = 1810.4 cm^–1^.^5^

As a first step toward obtaining laboratory spectral data on FeH^+^, we studied the FeH^+^ stretching mode by infrared multiple photon dissociation (IRMPD) via the argon tagging technique. Ar_*n*_FeH^+^ complexes are generated in a standard laser vaporization source^22−24^ and trapped at 80 K in a liquid-nitrogen cooled ion cyclotron resonance (ICR) cell^25^ where they are irradiated with light from a tunable optical parametric oscillator (OPO) system. ArFeH^+^ did not dissociate at the power of our tabletop laser system, probably due to the relatively high binding energy of the first argon atom and the limited density of states in the triatomic system. However, strong IRMPD signal was obtained with Ar_2_FeH^+^, reaction 3. Figure 1 shows the spectrum obtained in the 1600–2300 cm^–1^ region. One strong broad band lies at 1860 cm^–1^, and three weaker features are present at 2012 cm^–1^, 2054 cm^–1^, and 2078 cm^–1^. Gauss fits of the bands yield a full-width at half-maximum (fwhm) of 23 cm^–1^, 44 cm^–1^, 68 cm^–1^, and 46 cm^–1^, respectively:
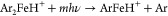
3

**Figure 1:**
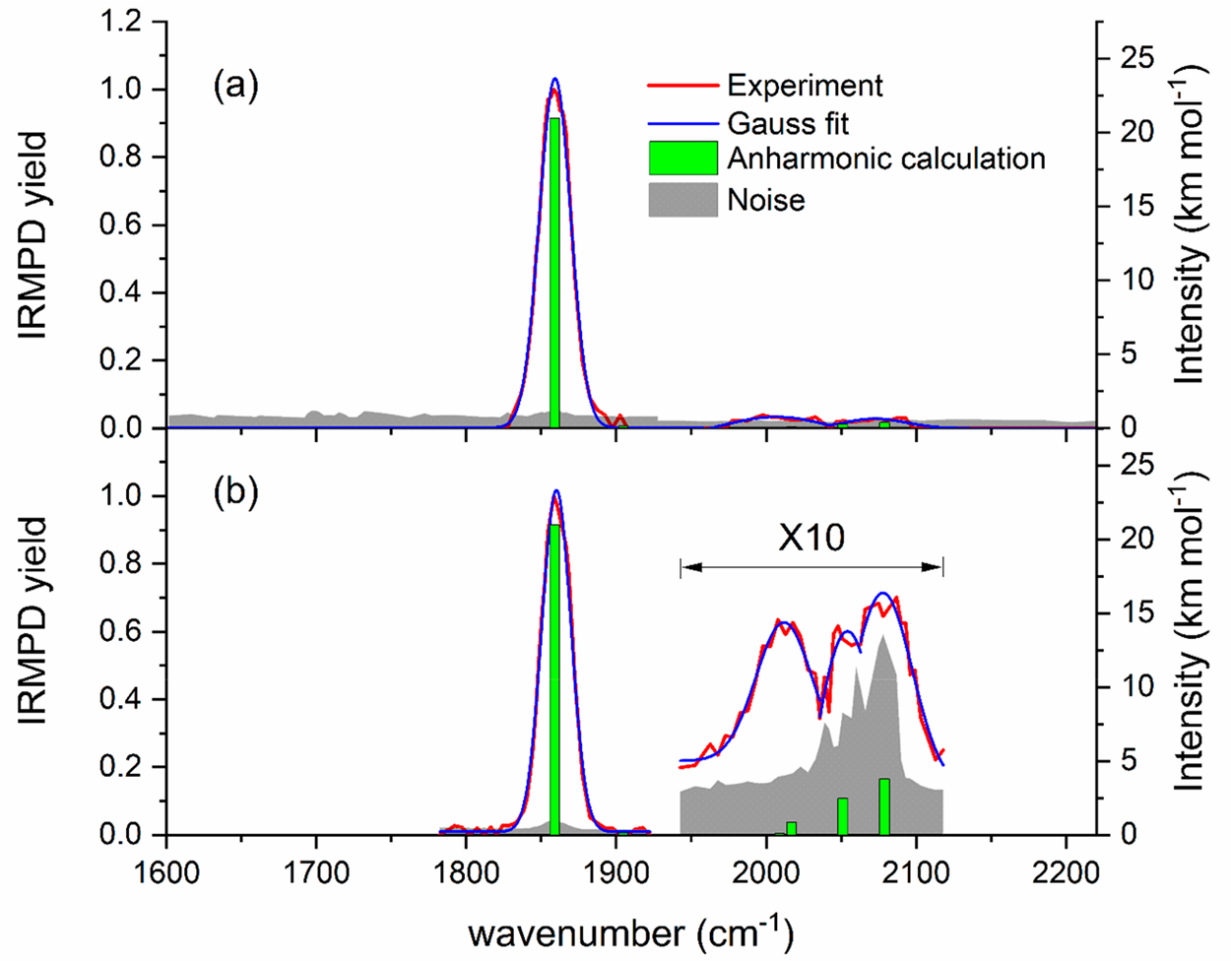
Experimental IRMPD spectrum of Ar_2_FeH^+^ at *T* ≈ 80 K. Anharmonic frequency analysis was performed at the B3LYP-D3/aug-cc-pVTZ level. (a) Precursor was trapped in the cooled cell for 3 s before isolation with 3 s time of irradiation. The main band lies at 1859 cm^–1^ with a 25 cm^–1^ fwhm. (b) Precursor was trapped in the cooled cell for 6 s with a 3 s time of irradiation in the 1780–1920 cm^–1^ region. The precursor was trapped in the cooled cell for 3 s with a 10 s time of irradiation for the upper 1940 cm^–1^. The main band lies at 1860 cm^–1^ with a 23 cm^–1^ fwhm. The spectrum above 1940 cm^–1^ is multiplied by a factor of 10 for better legibility.

To aid peak assignment, we performed geometry optimizations and frequency calculations of Ar_*n*_FeH^+^, *n* = 0–2, at the B3LYP-D3/aug-cc-pVTZ and CCSD/aug-cc-pVTZ levels of theory using B3LYP dispersion parameters as suggested by Grimme et al.^26,27^Figures 2 and S1 show the optimized structures and relative energies referenced to Ar_2_FeH^+^. The argon atoms preferentially bind to the metal center, which is advantageous for the present study since binding to the proton would lead to a stronger shift of the Fe–H stretching frequency. The calculated binding energy of the first and second argon atom is 45 kJ mol^–1^ and 29 kJ mol^–1^, respectively (CCSD/aug-cc-pVTZ), while the photon energy at the absorption maximum amounts to 22 kJ mol^–1^, less than the calculated binding energy. However, experimental IRMPD kinetics of Ar_2_FeH^+^ (Figures S2 and S3) exhibit first-order behavior and do not show any sign of an induction delay, which indicates that absorption of a single IR photon from the OPO causes dissociation. This suggests that the missing energy is present in the complex as vibrational energy. At room temperature, blackbody infrared radiative dissociation (BIRD)^28−32^ leads to a slow loss of one Ar atom, with a rate of 0.023 s^–1^, see Figure S2, while no fragmentation is detectable at a cell temperature of 80 K without laser irradiation. This observation confirms that energy exchange with the environment via blackbody radiation is efficient. The experimental IRMPD rate also depends on the temperature of the ICR cell, with 0.30 s^–1^ at 300 K and 0.13 s^–1^ at 80 K, Figure S3, clear evidence that IRMPD signal is obtained only from the fraction of the ion population that contains sufficient energy to be brought above the dissociation threshold by a single photon. Because of the small absorption intensity of 21.1 km mol^–1^ and low laser power in the range of 10 mW, however, the rate of photon absorption from the IR laser system is relatively small, which allows for significant radiative cooling before a second photon is absorbed. The internal energy distribution of the ions is determined by their initial internal energy upon leaving the ion source, absorption of ambient blackbody radiation, absorption of laser photons below the dissociation threshold, and radiative cooling. The first-order behavior of the IRMPD kinetics indicates that a stationary state of the internal energy distribution is reached quickly on the time scale of the experiment.

**Figure 2:**
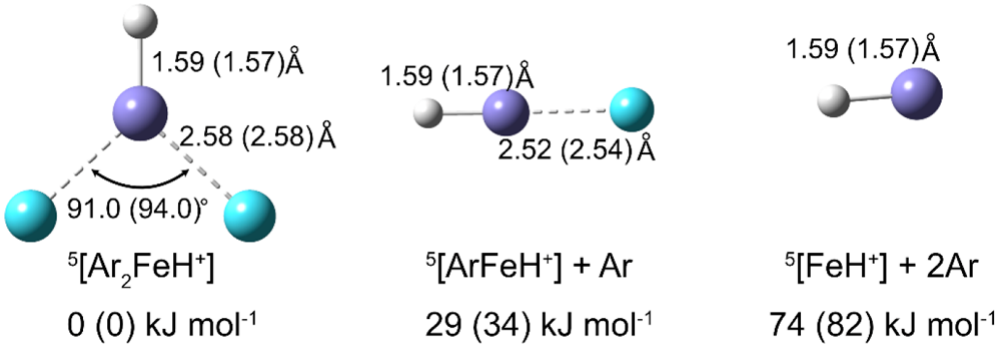
Structure of Ar_2_FeH^+^, ArFeH^+^, and FeH^+^ in quintet spin multiplicity along with zero-point corrected relative energies as calculated at the CCSD/aug-cc-pVTZ level. Geometry parameters and relative energies at the B3LYP-D3/aug-cc-pVTZ level are given in parentheses.

The calculated harmonic frequencies are summarized in Table 1, and benchmarking calculations are available in Tables S1–S4. They suggest that the intense band at 1860 cm^–1^ corresponds to the fundamental Fe–H stretch. Given that four vibrational modes lie around 148–221 cm^–1^, it seems plausible that the weaker features at higher energies are combination bands. To test this hypothesis, we performed anharmonic frequency analysis with three different density functionals and MP2 for comparison. B3LYP-D3 results are summarized in Table 2, listing unscaled frequencies and intensities for the Fe–H stretch fundamental ν̃_1_ and five combination bands ν̃_1_ + ν̃_*i*_ with the remaining modes *i* = 2–6; results for other methods are included in Table S4. A nearly perfect match of the fundamental mode is obtained with B3LYP-D3, with ν̃_1_ = 1862 cm^–1^ being only 2 cm^–1^ above the experimental band center. The calculated combination bands are plotted as vertical bars in Figure 1, and no Fermi resonances are predicted in the respective region by the anharmonic analysis. The calculated combination bands are in the right place, but the calculated intensities are relatively small, in relation to the Fe–H stretch peak. The lower intensity, however, may be compensated by the higher photon energy, which enlarges the ensemble of ions that have sufficient energy for dissociation after absorption of a single photon. If we assume that the anharmonic frequencies predicted on the B3LYP-D3/aug-cc-pVTZ level are realistic, we can assign all features to the combination bands listed in Table 2. Despite a thorough search, however, the ν̃_1_ + ν̃_6_ combination band with a slightly higher calculated IR intensity than ν̃_1_ + ν̃_4_ was not found experimentally, Figure S4. It should be noted that the different functionals we tested are not fully consistent with respect to the combination bands, Table S4; therefore, the assignment is not fully conclusive. While we cannot strictly rule out a contribution of low-lying electronically excited states to the weak features in the experimental spectrum, we consider this unlikely since the large-amplitude thermally excited motion of the Ar atoms should lead to much broader bands.

**Table 1:** Harmonic Frequencies ν̃ in Ar_2_FeH^+^ As Determined at the B3LYP+D3/aug-cc-pVTZ Level of Theory (Without Scaling), Irreducible Representations (irrep) Are Given within the C_2v_ Symmetry Group^a^

mode	irrep	vibration mode	ν̃/cm^–1^
ν̃_1_	A_1_	Fe–H stretch	1906
ν̃_2_	B_2_	Fe–H bend in the plane	234
ν̃_3_	B_1_	Fe–H bend out of the plane	206
ν̃_4_	A_1_	Fe–Ar symmetric stretch	157
ν̃_5_	B_2_	Fe–Ar asymmetric stretch	151
ν̃_6_	A_1_	Ar–Fe–Ar bend	46

a The molecule lies in the *yz* plane; the Fe–H bond is oriented along the *z* axis. Results for other methods are provided in the Supporting Information.

**Table 2:** Experimental Band Positions and fwhm for Ar_2_FeH^+^ Extracted from Figure 1b^a^

experiment		mode	theory		
Ar_2_FeH^+^	fwhm		Ar_2_FeH^+^	ArFeH^+^	FeH^+^
1860	23	ν̃_1_	1862 (21.07)	1878 (9.94)	1793 (1.89)
2078	46	ν̃_1_ + ν̃_2_	2090 (0.38)		
2054	68	ν̃_1_ + ν̃_3_	2061 (0.25)		
2012	44	ν̃_1_ + ν̃_4_	2014 (0.09)		
		ν̃_1_ + ν̃_5_	2005 (0.01)		
		ν̃_1_ + ν̃_6_	1908 (0.13)		

a Calculated vibrational frequencies (cm^–1^) and intensities (km mol^–1^, in parentheses) for Ar_2_FeH^+^, ArFeH^+^, and FeH^+^ in the Fe–H stretch region are given for comparison, calculated using anharmonic frequency analysis on the B3LYP-D3/aug-cc-pVTZ level. See the Supporting Information for benchmarking calculations.

The fwhm of the measured Fe–H stretch band is 23 cm^–1^. According to the laser beam analysis with a grating spectrometer, the laser line width is below 1 cm^–1^; thus, the observed fwhm is an inherent property of the studied ions. The thermal population of rotational levels at 80 K leads to a rotational envelope with 10 cm^–1^ fwhm, according to a simulation with pGopher,^33^ see Figure S5a. Thus, peak broadening of about 13 cm^–1^ needs to be accounted for from other sources, for example, thermal broadening. Looking at the list of vibrational frequencies in Table 1, it is evident that thermal population of vibrationally excited states is highest for the Ar–Fe–Ar bending mode ν̃_6_. At 80 K and for ν̃_6_ = 45.6 cm^–1^ (B3LYP/aug-cc-pVTZ), only 56.0% of the molecules are in their vibrational ground state *v*_6_ = 0, 24.6% are in *v*_6_ = 1, 10.9% in *v*_6_ = 2, and 4.8% in *v*_6_ = 3. Non-negligible 3.7% are in vibrational states *v*_6_ ≥ 4.

To find out whether thermal population of the Ar–Fe–Ar bending motion can explain the observed broadening, we performed a relaxed potential energy surface scan of the Ar–Fe–Ar angle. In variational transition state theory,^34^ it is well established that the density of states changes along the reaction pathway due to the change of the vibrational frequencies orthogonal to the reaction coordinate. At each fully converged geometry, we computed the projected frequencies^35^ for the remaining vibrational modes ν̃_*i*_, *i* = 1–5. The wavenumber of the Fe–H stretch ν̃_1_ is plotted in Figure 3 as a function of the Ar–Fe–Ar angle, together with the single-point energies of the relaxed scan. Using the vibrational energies in harmonic approximation, we can approximate the classical turning points of the vibration in each level, shown as vertical lines in Figure 3 and listed in Table S5. Since the Fe–H stretch oscillates 41-times faster than the Ar–Fe–Ar bend, the plot represents the frequency range of the Fe–H stretch vibration covered during the Ar–Fe–Ar vibration. This admittedly somewhat naïve approach gives a semiquantitative estimate of the broadening induced by thermal population of the Ar–Fe–Ar bending mode. For *v*_6_ = 2, the Ar–Fe–Ar angle ranges from 83.9° to 107.6°, which corresponds to Fe–H stretch frequencies from 1903.8 to 1889.1 cm^–1^, respectively, harmonic values without corrections. This amounts to a peak broadening of 14.7 cm^–1^. Using this value in a pGopher simulation as Gaussian broadening results in a 19 cm^–1^ wide unresolved band (Figure S5b), close to the experimental line width of 23 cm^–1^. This approach is all but exact, as the thermal population of other modes, especially of the symmetric and asymmetric Ar–Fe stretching modes, will also contribute. Nevertheless, the analysis strongly suggests that the thermal population of the Ar–Fe–Ar bending mode is the dominant contribution to the experimental line width of the Fe–H stretch.

**Figure 3:**
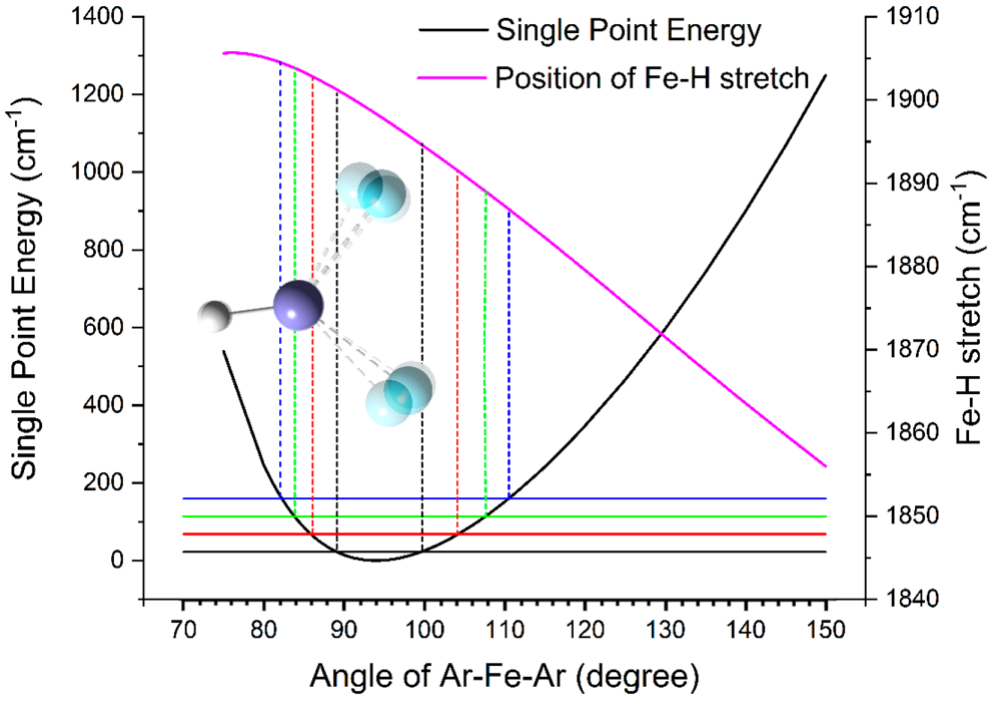
Single point energy for a relaxed scan of the Ar–Fe–Ar angle at the B3LYP-D3/aug-cc-pVTZ level and Fe–H stretch frequency calculated along the scan coordinate. Classical minimum and maximum angles for thermally populated vibrational states at *T* = 80 K are marked with dashed lines to guide the eye. The frequency of the Ar–Fe–Ar bend of 46 cm^–1^ was used, as calculated at the B3LYP-D3/aug-cc-pVTZ level within harmonic frequency analysis.

This analysis prompted us to look for experimental evidence for a thermal origin of the peak broadening. Table S6 shows the main band position and fwhm for different experimental conditions, and spectra are shown in Figure S6. The fwhm at room temperature is 30 cm^–1^, regardless of the time the ions are trapped in the ICR cell before irradiation. At 80 K, we start with 29 cm^–1^ fwhm for ions irradiated immediately after trapping. With longer trapping time in the ICR cell before irradiation, however, fwhm of the main peak is reduced to 25 and 23 cm^–1^ after 3 and 6 s delay before irradiation, respectively. This indicates that the ions are trapped with a vibrational temperature close to room temperature. When trapped in the ICR cell, the ions cool by emission of IR photons and slowly equilibrate with the cell walls, which reduces the line width. Moreover, there is a small but consistent redshift of the band position with increasing internal energy of the ions, which is consistent with the calculated frequency shift in Figure 3: for large Ar–Fe–Ar angles, the Fe–H stretch frequency change is more pronounced, and, at the same time, the system remains for a longer time at wide angles, since the potential is flatter than at the narrow angle turning point, which explains the observed redshift. The analysis in Figure 3 is thus fully consistent with the experimentally observed peak broadening and redshift, confirming the thermal origin of both effects.

Our experimental spectrum of the Fe–H stretching mode of Ar_2_FeH^+^ certainly does not provide the information required for the identification of the elusive FeH^+^ species in space. However, it provides the experimental data for benchmarking quantum chemical calculations, which is critical for accurate predictions. The anharmonic frequency analysis on the B3LYP-D3/aug-cc-pVTZ level of theory provided a close match with experiment. Employing this theory level to the untagged FeH^+^ molecular ion yields 1793 cm^–1^ (Table 2), 17 cm^–1^ below the 1810.4 cm^–1^ predicted by Cheng and DeYonker.^36^ In view of the large rotational constant of FeH^+^, *B*_*e*_ = 6.8766 cm^–1^,^5^ ro-vibrational lines are expected at a significant spacing and thus over a wide range. Considering Cheng and DeYonker’s prediction together with our experimental and theoretical results, we can conclude that the search for the vibration–rotation spectrum of FeH^+^ in the laboratory at interstellar temperatures of 50–100 K should focus on the region of 1750–1900 cm^–1^.

## Experimental and Computational Methods

IRMPD spectroscopy was performed on a modified Bruker Spectrospin CMS47X mass spectrometer described in detail before.^37,38^ The ICR cell is cooled with liquid nitrogen to 80 K to minimize the influence of blackbody radiation.^25^ Ar_2_FeH^+^ is obtained by laser vaporization of a solid iron target, using isotopically enriched ^56^Fe (STB Isotope Germany GmbH), followed by supersonic expansion in He seeded with 5% Ar and 5% H_2_. The ions are transferred to the ICR cell, trapped, mass selected, and irradiated for 1 s, followed by recording a mass spectrum. Tunable monochromatic infrared light is provided by an EKSPLA NT273-XIR OPO system operating at 1000 Hz pulse repetition rate. The wavelength was calibrated by a HighFinesse Laser Spectrum Analyzer IR-III, which also determined the line width as <1 cm^–1^.^39^

Quantum chemical calculations were performed using density functional theory (DFT) with various functionals, and detailed benchmarking including coupled cluster (CC) calculations can be found in the Supporting Information. Quintet spin multiplicity was used for Ar_*n*_FeH^+^ clusters, and very tight convergence criteria were used for optimization. Anharmonic frequency analysis was performed using numerical differentiation along vibrational modes. Wave function stability was tested prior to every calculation; stabilization of the wave function turned out to be crucial as electronically excited states were often obtained as a solution of the self-consistent field (SCF) calculation and prevented us from using codes in which wave function stabilization was not implemented. All calculations were performed in the Gaussian software package.^40^

## Supporting Information Available

IRMPD spectra at various temperatures and time delays, IRMPD kinetics; supplementary calculations: simulated ro-vibrational spectrum, benchmarking of computational approach, Cartesian coordinates and energies of all calculated structures (PDF)
Calibrated wavenumber, preirradiation delay and irradiation time, IRMPD yield and noise level of all data points in Figures 1 and S6 (XLSX)
