# Supplementary material for: Toward Detection of FeH+ in the Interstellar Medium: Infrared Multiple Photon Dissociation Spectroscopy of Ar2FeH+
Source: J Phys Chem Lett. 2022 Jun 21;13(25):5867–72. doi: 10.1021/acs.jpclett.2c01511 (PMC9251756; doi:10.1021/acs.jpclett.2c01511)
Supplement: Supplementary file 1 — jz2c01511_si_001.pdf [file jz2c01511_si_001.pdf]

Supporting Information

for

Towards Detection of FeH^+ in the Interstellar Medium: Infrared Multiple Photon Dissociation Spectroscopy of Ar_2FeH^+

Shan Jin, Jakob Heller, Christian van der Linde, Milan Ončák, and Martin K. Beyer**

Institut für Ionenphysik und Angewandte Physik, Universität Innsbruck, Technikerstrasse 25,

6020 Innsbruck, Austria

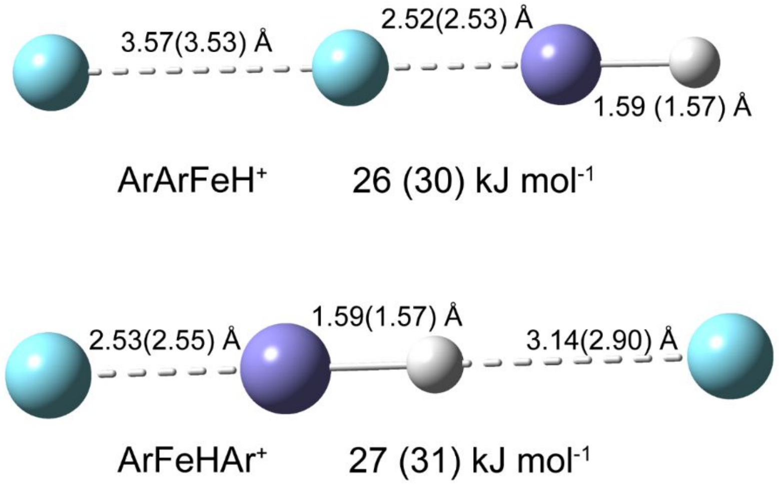

Figure S1. The conformers of Ar_2FeH^+ clusters with linear structure. Geometry parameters and energies relative to the Ar_2FeH^+ structure shown in Figure 2 were calculated at the CCSD/aug-cc-pVTZ level, B3LYP-D3/aug-cc-pVTZ values are given in the parentheses.

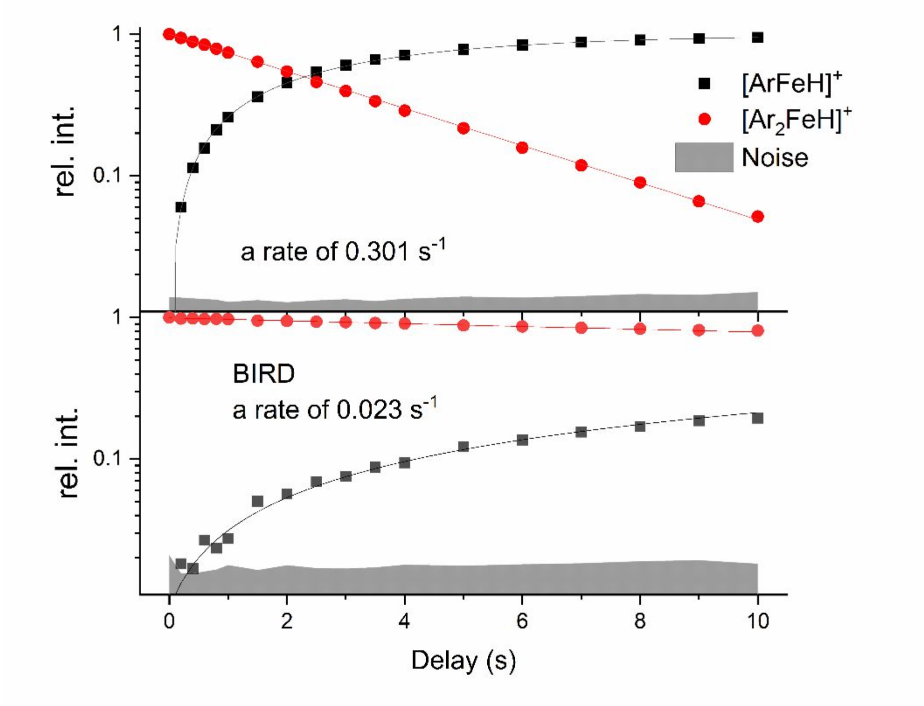

Figure S2. IRMPD kinetics at 1855 cm^{-1} (upper panel) and BIRD (lower panel) measured at room temperature.

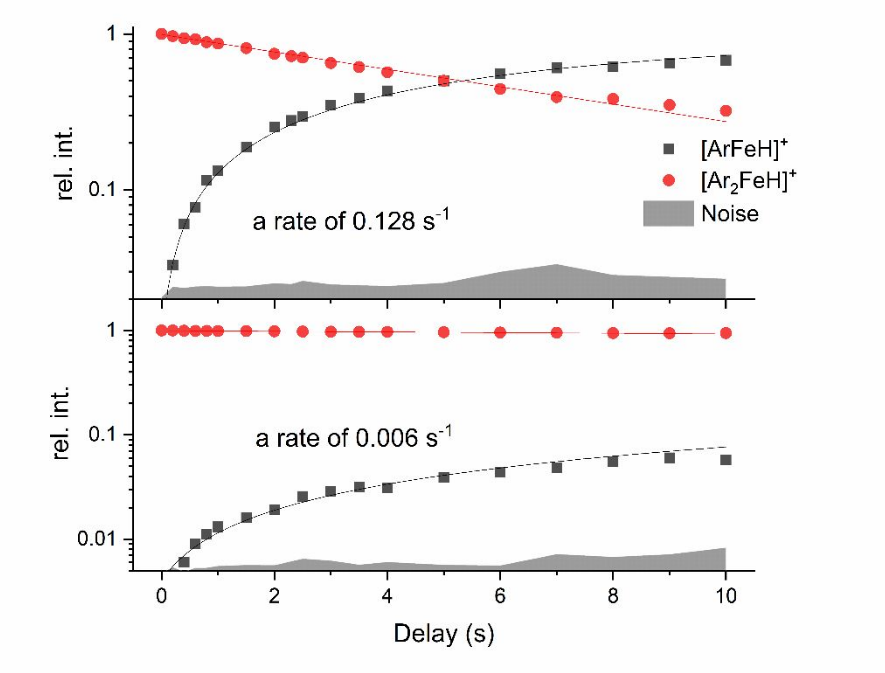

Figure S3. IRMPD kinetics at 1855 cm^{-1} (upper panel) and 2003 cm^{-1} (lower panel) measured at 80 K, fitted with a first-order rate law.

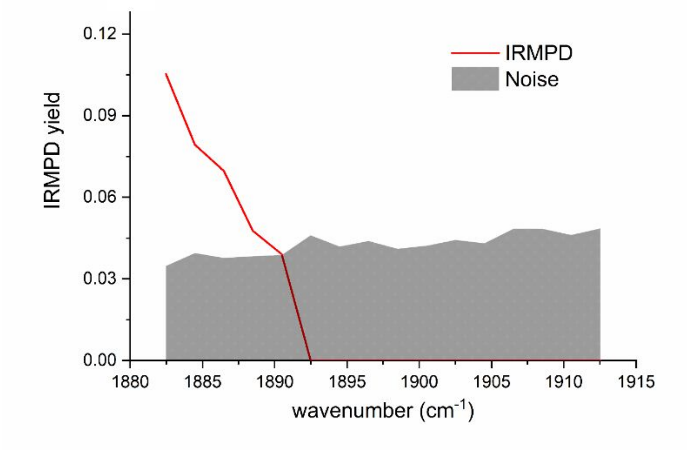

Figure S4. Experimental spectrum of Ar_2FeH^+ measured at 80 K. The precursor was trapped in the cooled cell for 3 s with a 10 s time of irradiation for the 1880 to 1910 cm^{-1} region. This spectrum shows no combinational band in this region. The signal at low wavenumbers is the tail of the main peak.

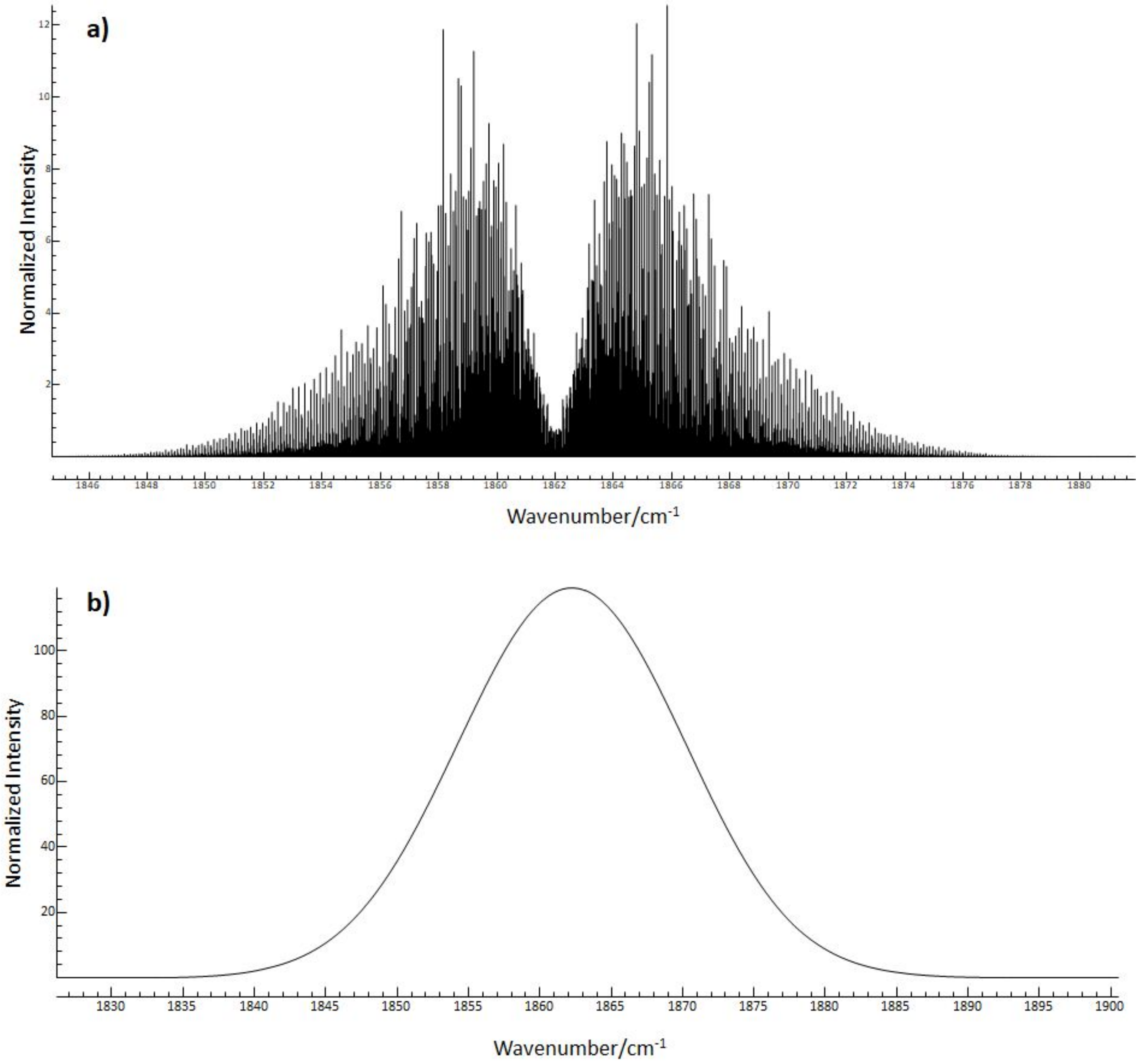

Figure S5. Ro-vibration spectrum of Ar_2FeH^+ simulated with pGopher at 80 K, assuming weak spin-orbit coupling. The molecular parameters were calculated at the B3LYP+D3/aug-cc-pVTZ level of theory within anharmonic vibrational analysis. a) No broadening; b) Gaussian broadening with FWHM of 14.7 cm^{-1}.

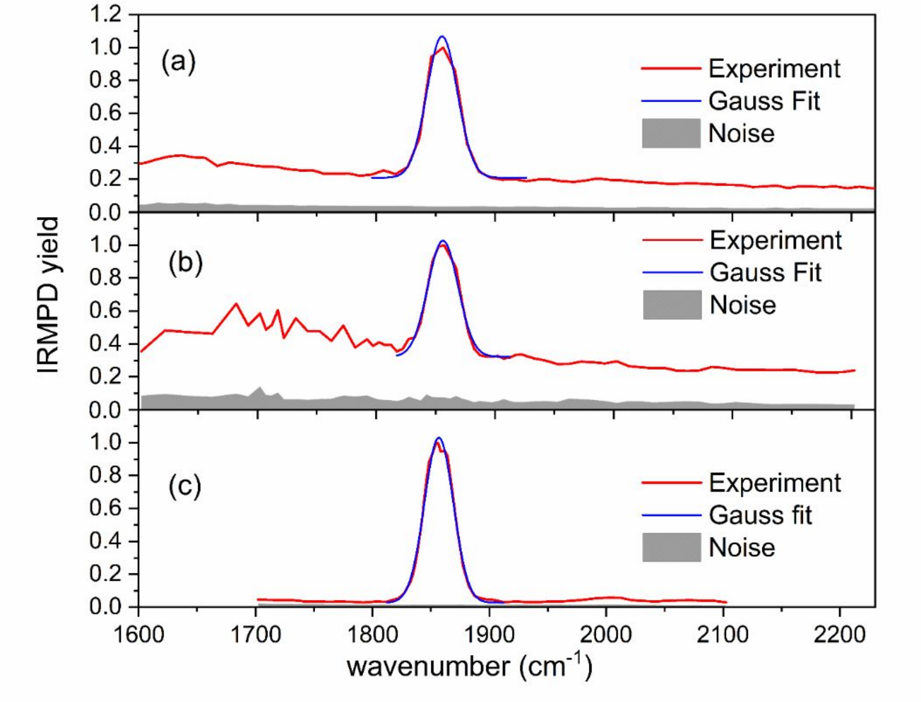

Figure S6. Experimental IRMPD spectra of Ar_2FeH^+, a) recorded at room temperature; b) recorded at room temperature, the precursor is stored in the ICR cell for 2 s before isolation; c) is recorded at 80 K. The main band was fitted by a Gaussian function at 1856 cm^{-1} (a), 1856 cm^{-1} (b), 1857 cm^{-1} (c) with FWHM of 30 cm^{-1}, 30 cm^{-1} and 29 cm^{-1}, respectively.

Table S1. Harmonic frequencies of Fe-H stretching (in cm^{-1}) for FeH^+ calculated using various methods and two different basis sets. The frequencies are unscaled.

Basis	B3LYP	B3LYP-D3	BMK	CAM-B3LYP	*ω*B97XD	MP2	CCSD
aug-cc-pVDZ	1878	1855	1885	1926	1924	1943	1865
aug-cc-pVTZ	1879	1855	1894	1928	1924	1952	1871

Table S2. Ar_2FeH^+ harmonic vibrational frequencies (in cm^{-1}) as calculated using various methods and the aug-cc-pVDZ basis set. The frequencies are unscaled.

mode	B3LYP	B3LYP-D3	BMK	CAM-B3LYP	*ω*B97XD	MP2	CCSD
Fe-Ar bend	42	45	44	45	45	53	51
Fe-Ar asymmetry stretch	141	144	95	151	148	131	133
Fe-Ar symmetry stretch	148	153	127	160	155	154	151
Fe-H bend out of the plane	206	206	235	214	202	212	211
Fe-H bend in the plane	225	222	227	231	221	204	213
Fe-H stretch	1905	1905	1924	1946	1930	1923	1902

Table S3. Ar_2FeH^+ harmonic vibrational frequencies (in cm^{-1}) as calculated using various methods and the aug-cc-pVTZ basis set. The frequencies are unscaled.

mode	B3LYP	B3LYP-D3	BMK	CAM-B3LYP	*ω*B97XD	MP2	CCSD
Fe-Ar bend	43	46	48	45	47	54	51
Fe-Ar asymmetry stretch	148	151	174	160	146	147	148
Fe-Ar symmetry stretch	152	157	186	165	151	166	161
Fe-H bend out of the plane	207	206	238	214	207	209	209
Fe-H bend in the plane	237	234	227	245	236	211	221
Fe-H stretch	1906	1906	1927	1947	1931	1924	1901

Table S4. Vibrational frequencies (cm^{-1}) and intensity ($km\ mol^{-1}$) of Ar_2FeH^+ in the Fe-H stretch region, calculated with anharmonic frequency analysis. The aug-cc-pVTZ basis set was employed.

mode	B3LYP		CAM-B3LYP		*ω*B97XD		MP2	
	Frequency	Intensity	Frequency	Intensity	Frequency	Intensity	Frequency	Intensity
ν1	1869	20.04	1919	39.99	1986	38.35	1879	87.92
ν1+ν2	2099	0.34	2206	0.73	2223	0.71	2150	1.41
ν1+ν3	2069	0.23	2185	0.50	2275	0.49	2143	1.06
ν1+ν4	2016	0.08	2080	0.08	2185	0.08	2038	0.14
ν1+ν5	2010	0.01	2075	0.02	2198	0.02	2107	0.29
ν1+ν6	1914	0.16	1971	0.18	2009	0.17	1933	0.00

Table S5. Fe-H stretch frequency at minimum and maximum classical angle of the four lowest lying, thermally populated vibrational level of the Ar-Fe-Ar bending mode, together with the resulting line broadening.

Vibrational level	Energy (cm^{-1})	Thermal population T = 80 K	Classical minimum and maximum angle (degree)	Frequency (cm^{-1})	Line broadening (cm^{-1})
$v = 0$	22.8	0.560	89.1<br>99.7	1901.3<br>1894.4	6.9
$v = 1$	68.4	0.246	85.9<br>104.3	1903.0<br>1891.5	11.5
$v = 2$	114.0	0.109	83.9<br>107.6	1903.8<br>1889.1	14.7
$v = 3$	159.6	0.048	82.3<br>110.5	1904.4<br>1886.9	17.5

Table S6. Band position and full width at half maximum (FWHM) at room temperature and 80 K, accompanied by a delay of 0~6 s before infrared irradiation.

exp. Temperature	exp. setup	band position / cm^{-1}	FWHM / cm^{-1}
room temperature	delay 0 s, t_{ir} = 3 s	1856	30.4
	delay 2 s, t_{ir} = 3 s	1856	30.0
80 K	delay 0 s, t_{ir} = 3 s	1857	29.2
	delay 3 s, t_{ir} = 3 s	1859	24.7
	delay 6 s, t_{ir} = 3 s	1860	22.8

Cartesian coordinates (Å) of optimized structures along with their electronic energy (Hartree)

FeH+, B3LYP/aug-cc-pVDZ
E = −1264.020617
H 0.000000 0.000000 −1.516234
Fe 0.000000 0.000000 0.058317

FeH+, B3LYP/aug-cc-pVTZ
E = −1264.027137
H 0.000000 0.000000 −1.514981
Fe 0.000000 0.000000 0.058268

FeH+, B3LYP+D3/aug-cc-pVDZ
E = −1264.021389
H 0.000000 0.000000 −1.517485
Fe 0.000000 0.000000 0.058365

FeH+, B3LYP+D3/aug-cc-pVDZ
E = −1264.027908
H 0.000000 0.000000 −1.516206
Fe 0.000000 0.000000 0.058316

FeH+, BMK/aug-cc-pVDZ
E = −1263.434755
H 0.000000 0.000000 −1.529625
Fe 0.000000 0.000000 0.058832

FeH+, BMK/aug-cc-pVTZ
E = −1263.441314
H 0.000000 0.000000 −1.527366
Fe 0.000000 0.000000 0.058745

FeH+, CAM-B3LYP/aug-cc-pVDZ
E = −1264.052529
H 0.000000 0.000000 −1.509511
Fe 0.000000 0.000000 0.058058

FeH+, CAM-B3LYP/aug-cc-pVTZ
E = −1264.059099
H 0.000000 0.000000 −1.508126
Fe 0.000000 0.000000 0.058005

FeH+, wB97XD/aug-cc-pVDZ
E = −1264.002942
H 0.000000 0.000000 −1.514707
Fe 0.000000 0.000000 0.058258

FeH+, wB97XD/aug-cc-pVTZ
E = −1264.008413
H 0.000000 0.000000 −1.513280
Fe 0.000000 0.000000 0.058203

FeH+, MP2/aug-cc-pVDZ
E = −1262.899474
H 0.000000 0.000000 −1.530711
Fe 0.000000 0.000000 0.058874

FeH+, MP2/aug-cc-pVTZ
E = −1262.934144
H 0.000000 0.000000 −1.523985
Fe 0.000000 0.000000 0.058615

FeH+, CCSD/aug-cc-pVDZ
E = −1262.916710
H 0.000000 0.000000 −1.537590
Fe 0.000000 0.000000 0.059138

FeH+, CCSD/aug-cc-pVTZ
E = −1262.949248
H 0.000000 0.000000 −1.532187
Fe 0.000000 0.000000 0.058930

Ar, B3LYP/aug-cc-pVTZ
ar.b3lyp.com.log
E = -527.560001974
Ar 0.000000 0.000000 0.000000

Ar, CCSD/aug-cc-pVTZ
E = -527.04141055
Ar 0.000000 0.000000 0.000000

FeHAr+, B3LYP+D3/aug-cc-pVTZ
E = −1791.606155 −1791.606155
H 0.000000 0.000000 2.551693
Fe 0.000000 0.000000 0.981841
Ar 0.000000 0.000000 −1.559975

FeHAr+, CCSD/aug-cc-pVTZ
E = −1790.007667
H 0.000000 0.000000 2.564964
Fe 0.000000 0.000000 0.974161
Ar 0.000000 0.000000 −1.549619

ArArFeH+, B3LYP+D3/aug-cc-pVTZ
E = -2319.167466
H 0.000000 0.000000 3.999903
Fe 0.000000 0.000000 2.430177
Ar 0.000000 0.000000 -0.100499
Ar 0.000000 0.000000 -3.631973

ArArFeH+, CCSD/aug-cc-pVTZ
E = -2317.050237
H -0.000000 0.000000 -4.022215
Fe -0.000000 0.000000 -2.431198
Ar 0.000000 -0.000000 0.083821
Ar 0.000000 -0.000000 3.651366

ArFeHAr+, B3LYP+D3/aug-cc-pVTZ
E = -2319.167238
Ar 0.000000 0.000000 3.121731
Fe 0.000000 0.000000 0.575375
H 0.000000 0.000000 -0.993409
Ar 0.000000 0.000000 -3.897639

ArFeHAr+, CCSD/aug-cc-pVTZ
E = -2317.049820
Ar -0.000000 0.000000 -3.181254
Fe -0.000000 0.000000 -0.655730
H 0.000000 -0.000000 0.934534
Ar 0.000000 -0.000000 4.076500

FeHAr2+, B3LYP/aug-cc-pVDZ
E = -2319.136624
H 0.000000 0.000000 2.556163
Fe 0.000000 0.000000 0.979633
Ar 0.000000 1.929772 -0.778517
Ar -0.000000 −1.929772 -0.778517

FeHAr2+, B3LYP/aug-cc-pVTZ
E = -2319.173705
H -0.000000 -0.000000 2.534304
Fe -0.000000 -0.000000 0.958732
Ar -0.000000 1.929069 -0.762815
Ar -0.000000 –1.929069 -0.762815

FeHAr2+, B3LYP+D3/aug-cc-pVDZ
E = -2319.141922
h -0.000000 -0.000000 2.577185
fe -0.000000 -0.000000 1.001415
ar 0.000000 1.882488 -0.794833
ar -0.000000 –1.882488 -0.794833

FeHAr2+, B3LYP+D3/aug-cc-pVTZ
E = -2319.179026
H -0.000000 0.000000 2.554347
Fe -0.000000 0.000000 0.979564
Ar -0.000000 1.885072 -0.778417
Ar -0.000000 –1.885072 -0.778417

FeHAr2+, BMK/aug-cc-pVDZ
E = -2318.234223
H 0.000000 0.000000 2.588055
Fe 0.000000 0.000000 0.995584
Ar -0.000000 1.866862 -0.790923
Ar -0.000000 –1.866862 -0.790923

FeHAr2+, BMK/aug-cc-pVTZ
E = -2318.269822
H -0.000000 -0.000000 2.561886
Fe -0.000000 -0.000000 0.971145
Ar 0.000000 1.866208 -0.772546
Ar -0.000000 -1.866208 -0.772546

FeHAr2+, CAM-B3LYP/aug-cc-pVDZ
E = -2319.217319
H -0.000000 0.000000 2.521829
Fe -0.000000 -0.000000 0.951541
Ar 0.000000 1.886192 -0.757275
Ar -0.000000 –1.886192 -0.757275

FeHAr2+, CAM-B3LYP/aug-cc-pVTZ
E = -2319.179622
H 0.000000 0.000000 2.547172
Fe 0.000000 0.000000 0.975982
Ar -0.000000 1.881200 -0.775630
Ar -0.000000 -1.881200 -0.775630

FeHAr2+, wB97XD/aug-cc-pVDZ
E = -2319.091317
H 0.000000 0.000000 2.558890
Fe 0.000000 0.000000 0.980494
Ar 0.000000 1.900355 -0.779215
Ar -0.000000 –1.900355 -0.779215

FeHAr2+, wB97XD/aug-cc-pVTZ
E = -2319.128864
H 0.000000 0.000000 2.529388
Fe 0.000000 0.000000 0.951798
Ar -0.000000 1.906600 -0.757671
Ar -0.000000 –1.906600 -0.757671

FeHAr2+, MP2/aug-cc-pVDZ
E = -2316.837791
h 0.000000 -0.000000 2.635495
fe 0.000000 -0.000000 1.038651
ar -0.000000 1.829937 -0.823345
ar -0.000000 -1.829937 -0.823345

FeHAr2+, MP2/aug-cc-pVTZ
E = -2317.012920
H 0.000000 0.000000 2.607295
Fe 0.000000 0.000000 1.016064
Ar -0.000000 1.808175 -0.806249
Ar -0.000000 -1.808175 -0.806249

FeHAr2+, CCSD/aug-cc-pVDZ
E = -2316.877389
h 0.000000 0.000000 2.626651
fe 0.000000 0.000000 1.028968
ar -0.000000 1.854476 -0.816106
ar -0.000000 –1.854476 -0.816106

FeHAr2+, CCSD/aug-cc-pVTZ
E = -2317.060105
h -0.000000 0.000000 2.600158
fe -0.000000 0.000000 1.006819
ar 0.000000 1.838278 -0.799373
ar -0.000000 -1.838278 -0.799373
